# Healthy Lifestyle and Academic Performance in Middle School Students from the Region of Aragón (Spain)

**DOI:** 10.3390/ijerph18168624

**Published:** 2021-08-15

**Authors:** Beatriz Sánchez-Hernando, Isabel Antón-Solanas, Raúl Juárez-Vela, Vicente Gea-Caballero, María Inmaculada Carboneres-Tafaner, Elisa Ferrer-Gracia, Javier Gállego-Diéguez, Iván Santolalla-Arnedo, Ángel Gasch-Gallén

**Affiliations:** 1 Health Center “Amparo Poch”, Aragón Health Care System ( SALUD), C/Emilia Pardo Bazán, s/n, 50018 Zaragoza, Spain; beasanhern@gmail.com; 2 Nursing Research Group in Primary Care of Aragon (GIIS094-GENIAPA), Aragón Health Research Institute, Avda. San Juan Bosco 13, 50009 Zaragoza, Spain; ianton@unizar.es (I.A.-S.); angelgasch@unizar.es (Á.G.-G.); 3 Department of Physiatry and Nursing, University of Zaragoza, C/Domingo Miral s/n, 50009 Zaragoza, Spain; 4 Department of Nursing, Biomedical Research of La Rioja, University of La Rioja, CIBIR C/Duquesa de la Victoria 88, 26004 Logroño, La Rioja, Spain; ivan.santolalla@unirioja.es; 5 Nursing School La Fe, Adscript Center of Universidad de Valencia, Research Group GREIACC, Health Research Institute La Fe, Avda. Fernando Abril Martorell 106, 46026 Valencia, Spain; 6 Generalitat Valenciana, CEIP Mare de Deu de la Vallivana, Conselleria de Educació, C/Carles Albors 18, Picassent, 46220 Valencia, Spain; carboneres_martaf@gva.es; 7 Health Promotion Section, General Direction of Public Health, Government of Aragon, Vía Universitas 36, 50017 Zaragoza, Spain; eferrerg@aragon.es; 8 Head of the Information, Transparency and Participation Service, Health Department, Government of Aragon, Vía Universitas 36, 50017 Zaragoza, Spain; jgallego@aragon.es

**Keywords:** adolescent, habits, academic performance, diet, sleep, exercise, social media, substance-related disorders

## Abstract

A healthy lifestyle is important to the present and future development and health of school age people. This study aims to analyze the relationship between daily lifestyle habits and academic performance in a sample of adolescents from the autonomous community of Aragon (Spain). We performed a cross-sectional study to analyze the lifestyle habits and academic performance of a total of 1745 7th and 8th grade middle school students during the academic year 2018–2019; the participants were selected from a random sample of 43 middle schools from the region of Aragon. The following data were collected through an anonymized, previously validated questionnaire: diet, sleep, physical activity, use of screens, use of toxic substances, and academic performance. We found a statistically significant association between all the lifestyle habits analyzed and academic performance (*p* < 0.001) in our sample. Based on our findings, we suggest that health promotion and education in healthy lifestyles should be integrated in middle school curricula to improve academic performance and, more importantly, to promote both present and future health outcomes of adolescents.

## 1. Introduction

The educational sphere is becoming increasingly aware of the influence it exerts on its students, not only at the educational level but also on their present and future health. More and more educational centers are integrating educational strategies which are based on transversal salutogenic approaches [1,2]. In this sense, health-promoting schools (HPS) are an example of multifactorial intervention in the educational environment [3,4], in which interventions on social determinants of health and the promotion and development of basic competencies are implemented [5]. This is important due to the potential effect of healthy lifestyles on academic performance [6].

For instance, maintaining a balanced diet and practicing regular physical exercise leads to improved health outcomes in the short, medium, and long term, and decreases the risk of suffering cardiovascular and other chronic conditions, improving both physical and mental health. These habits are beneficial for the general population, but especially for children and adolescents [7,8]. In addition, previous studies have reported a positive association between maintaining a healthy diet and practicing regular physical exercise on academic performance [9,10,11,12,13]. However, some studies have reported an inadequate dietary intake in this population, including insufficient consumption of fruit and vegetables, and an excessive consumption of meat, sweets, and sugary drinks [14,15]. Other factors contributing to a less than optimal lifestyle in this population include digital transformation, which has increased the use of screens and, subsequently, reduced daily physical activity [14]. Consequently, childhood and adolescent obesity is becoming a serious public health issue worldwide [16].

Another factor that could influence academic performance is maintaining an adequate and sufficient sleep pattern. Some studies have reported a positive association with academic performance [17,18].

In contrast, excessive use of electronic devices [19], and use of tobacco, alcohol, and other substances [20,21,22] could negatively affect academic performance. Adolescence is critical risk period for the initiation of substance use including tobacco, alcohol, and other substances, which have a devastating effect on the body, especially in young people [23].

However, the evidence on the association between lifestyle and academic performance is not clear. On the one hand, some studies have observed weak or non-significant associations between diet and academic performance [9,15,24,25]. On the other hand, previous studies have not found an association between regular physical exercise and academic performance [12,15,26]. In addition, the evidence available is limited as there are no previous studies that analyze these variables simultaneously.

Thus, it is important to investigate the relationship between maintaining a healthy lifestyle and academic performance in adolescents, as it may impact on policies, decisions and interventions designed and implemented by educational institutions in terms of promoting the students’ health and, thus, improving their academic performance. Therefore, we aimed to analyze the association between specific lifestyle habits, namely diet, sleep, physical activity, use of screens, and use of substances, and academic performance in adolescents aged 12–15 from the region of Aragón, Spain.

## 2. Materials and Methods

### 2.1. Design

A cross-sectional study was designed and carried out during the 2018–2019 academic year.

### 2.2. Sampling and Study Population

We performed a two-stage sampling strategy. First, we randomly selected 43 out of a total of 185 middle schools from the region of Aragón, Spain, taking into account the characteristics of the educational centers according to whether they were rural/urban, private/public and HPS/non-HPS centers. The random sample of schools was selected using the application “Research Randomizer” (www.randomizer.org). Subsequently, we invited all the 7th and 8th grade students registered at the selected middle schools (*N* = 5132). We excluded all the students who could not speak Spanish. Finally, a total sample of 1745 7th and 8th grade middle school students accepted to participate in this study. The minimum significant sample we needed was 379 students (out of a total census of 27,184), so this sample was representative.

### 2.3. Data Collection

The participants completed a self-administered, anonymous questionnaire during April 2019. This tool was adapted from the instrument implemented in the Health Behavior in School-Age Children (HBSC) Spain 2014 study, and subsequently validated [14]. The adaptation of the self-administered questionnaire was carried out using the expert panel technique [27]. We recruited six experts who met the following requirements: (a) being a qualified nurse or a physician, (b) having a minimum work experience of 5 years in public and/or community health, (c) having demonstrable participation in at least 5 projects with HPS. A total of 6 experts were recruited, who took part in two sessions, lasting approximately 90 min each, in December 2017. Internal consistency of the adapted version of the questionnaire (Cronbach’s alpha) was 0.85. Factorial analysis explained 75.25% of the model variance. The instrument’s validity was evaluated using different indicators (NFI = 0.802; RMSEA = 0.067; CFI = 0.891; SRMR = 0.093) and showed good adjustment. Mainly, the Promax oblique rotation was used with the objective of analyze by means the principal components to precisely reflect the interaction between the elements according to different bibliography [28,29]. For the model’s degree of fit, the Root Mean Standard Error (RMSEA) was also obtained as well as the Standardized Root Mean Square Residual (SRMR), which must be below 0.08 according to [30]. The rest of analysis was completed according to different publications [30,31,32].

### 2.4. Variables

The adapted version of the instrument comprised 42 items classified into 7 subscales, namely, sociodemographic characteristics (4 items), diet (9 items), sleep (1 item), physical activity (4 items), use of screens (6 items), substance use (17 items), and academic performance (1 item).

The sociodemographic characteristics included the following variables: sex, age, year of study by age and level of perceived health.

The following variables were included in the diet subscale: breakfast during the week (yes; no), weekly consumption of fruit and vegetables, chips or salty snacks, sweets, soft drinks or sugary drinks, meat, fish, and milk or dairy products (up to once per week; 2 to 6 times a week; at least once a day).

The sleep subscale included the following items: hours of nighttime rest during the week (less than 7 h; from 7 to 9 h; more than 9 h).

The items included in the physical activity subscale were: weekly frequency of physical activity during leisure time (never to once a month; 1 to 3 times a week; 4 to 7 times a week), number of hours of physical activity during leisure time a week (1 h or less; 2 to 3 h; more than 4 h), playing or practicing team sports and physical activities (less than 3 times a month; 1 to 3 times a week), practicing individual physical activities (less than 3 times a month; 1 to 3 times a week).

The subscale screen use included the following items: time spent daily playing games, time spent daily watching TV, videos, and other displays on a screen, and time spent daily using screens for homework or use of social networks (weekdays and weekends) (less than 2 h; 2 h; more than 2 h).

Finally, the subscale substance use included the following items: use of tobacco (yes; no), use of alcohol, including wine, mixed alcoholic drinks, liquor shots, and other beverages (never; rarely; daily-monthly), use of other substances including cocaine, hashish or marijuana, ecstasy or pills, amphetamines or speed, non-prescription drugs, LSD, glue or solvents, other drugs (never; at least once), age of tobacco use onset (never; younger than 11 to older than 14 years), age of alcohol use onset (never; before 13; after 13), age of first binge-drinking episode (never; before 13; after 13), age of hashish or marijuana use onset in the form of a joint (never; from less than 11 to more than 14 years). The students’ academic performance was assessed using the students’ average score for the whole academic year; in Spain, an academic score is a number ranging from 0 to 10.

### 2.5. Data Analysis

Categorical variables were analyzed using frequencies and percentages; numerical variables were analyzed using mean and standard deviation. The Kolmogorov–Smirnov and Shapiro–Wilk tests were used to assess normality. A correlation study was performed between the different lifestyle habits measured and academic performance using Kendall’s Tau B test. For the inferential analysis, we used the Kruskal–Wallis test, except for those items measured through a dichotomous response; in these cases, we used the Mann–Whitney U test. Statistical analyses were completed using the software STATA/SE v16.0. (StataCorp. College Station, TX, USA).

### 2.6. Ethical Considerations

All the participants, as well as a parent or a legal tutor, gave their written consent to take part in this study. All the data collected were anonymized and confidentially was guaranteed according to the Data Protection Regulation (EU) 2016/679 of the European Parliament and the Spanish Organic Law 3/2018. Participation in this study was voluntary. No ethical, moral, or legal conflict was declared by any of the participants, who did not receive any form of financial or other compensation for taking part in this investigation. This project was endorsed by the General Directorate of Public Health and the Direction of Innovation, Equity, and Participation of the Government of Aragon.

## 3. Results

A total of 1745 students completed the questionnaire (response rate 34%). Mean age was 13.03 years (SD ± 0.82; range 12–16); 45.43% of our respondents were male. More than 80% of our sample were enrolled in the right course for their age. More than 90% of the students declared to be in good or excellent health. In terms of their academic performance, the student’s average grade was 6.75 out of 10.

We analyzed the relationship between the various lifestyle habits and academic performance in our sample. Statistically significant correlations between healthy dietary habits and academic performance were observed (Table 1). Specifically, we identified a positive correlation between eating breakfast, consuming fruit, vegetables, fish, and milk or dairy products and academic performance, whereas a negative correlation was found between consuming chips, sweets, and soft drinks, and academic performance. In the inferential study, better academic results were observed when breakfast was eaten during the week, fruit and vegetables were consumed at least once a day and fish and milk were consumed 2–6 times a week. In addition, the higher the consumption of chips, sweets, and soft drinks, the lower the academic performance (*p* < 0.001).

A statistically significant and positive correlation was observed between sleep patterns and academic performance (*p* = 0.000) (Table 2); that is, the more hours of sleep at night, the better the student’s academic performance.

Statistically significant and positive correlations were observed between frequency of weekly physical activity, hours of weekly physical activity, team and individual physical activity, and academic performance (Table 3). Further, a statistically significant association was found between academic performance and physical activity in terms of weekly frequency, hours per week, and individual activity.

A negative correlation was observed between use of screens and academic performance. A statistically significant association was found between use of screens and academic performance (*p* < 0.001) (Table 4). We observed that the greater the use of screens during the week, in the three modalities analyzed, the poorer the students’ academic performance.

A negative correlation was observed between use of substances and age of onset, and academic performance. A statistically significant association was observed between the consumption of tobacco, alcohol, and other substances, and academic performance; a statistically significant and negative relationship was also observed in terms of age of onset of use of substances and academic performance (Table 5).

## 4. Discussion

This study aimed to analyze the relationship between different lifestyle habits (diet, sleep, physical exercise, use of screens, and consumption of toxic substances) and academic performance in 7th and 8th grade middle school students.

The evidence suggests that maintaining a healthy diet during childhood and adolescence helps to prevent cardiovascular and other chronic conditions in the short, medium, and long-term [7]. However, less in known about the relationship between dietary habits and academic performance in this population. Our findings suggest that a significant association exists between eating breakfast regularly and academic performance. This is in agreement with previous studies [24,33] that reported that those adolescents who ate breakfast regularly had better cognitive capacity and achieved better academic results. We also found that there is a positive association between consuming healthy foods, such as fruit, vegetables, and fish, and academic performance. This is in line with previous studies [6,10,11,24] that established and association between adherence to healthy eating styles, like the Mediterranean diet, and academic success. Interestingly, just as eating healthy foods was associated with better academic performance, eating unhealthy foods was associated with worse academic results. For instance, consuming soft drinks, salty and sugary snacks was linked to worse academic performance in our study, and in a previous study [10] in a similar population. We did not find a significant association between the consumption of meat and academic performance in our sample. Our findings regarding the relationship between dietary habits and academic performance differ from those obtained in previous studies [9,15,25,34], which did not find an association between the two. We hope that our study, carried out on a large sample of adolescents from different middle schools, may contribute to supporting the argument that a positive association exists between dietary habits and academic performance.

Regarding the adolescents’ sleep pattern, it is well known that lack of sleep can reduce cognitive abilities [17], and thus reduce the possibilities of academic success in this population. Not surprisingly, we found a positive association between the number of hours of sleep at night and academic performance, as confirmed in previous studies [17,35].

Physical exercise is beneficial for health. In fact, physical activity is frequently used as prevention and treatment of a wide range of health problems including obesity and cardiovascular risk factors, among others [36]. At a cognitive level, physical activity can improve brain activity and foster the development of children’s cognition [37,38]. We found a significant association between academic performance and weekly frequency of physical activity, number of weekly hours of physical activity during leisure time and practicing physical exercise individually. Similar findings were obtained by other authors [6,39]. However, the evidence is not clear. A previous review did find a positive association between physical exercise and academic performance, but with a low signification [36]. Additionally, some of the studies that did confirm the association between the two, limited their recommendations to the performance of a specific activity [12,35]. Other authors [9] also confirmed the association between exercise and academic performance, but only in specific courses from the curriculum, and other studies were unable to find a relationship between them [15]. Our findings suggest that practicing physical activity individually leads to improved academic results. However, we were unable to demonstrate an association between practicing team sports and academic performance. This suggests that, despite the multiple benefits of practicing sports, both as a team and individually options, it is necessary to continue research to go deeply into its possible effect on academic performance in adolescents.

Regarding the use of screens, it has been observed that excessive use beyond the recommendations is related to a sedentary lifestyle and a decrease in physical exercise during leisure time [19,40]. This may increase the risk of becoming overweight or obese, and it may negatively influence academic performance. We observed a significant relationship between use of screens during the week and academic performance. Our findings are confirmed by other authors [41], who suggested that inappropriate and excessive use of screens could lead to learning difficulties in both children and adolescents. In addition, we observed a significant association between use of screens during the weekend and academic performance, yet the relationship between them was not lineal, which greatly complicates the interpretation of our findings. We argue that this may be due to several factors. For instance, use of screens during the weekend tends to be higher generally. That is, both the students who used screens excessively during the week, and those who did not, spent too much time sitting in front of a screen during the week. This may explain why fewer differences were found between both groups during the weekend.

It is well known that using certain substances can decrease learning and memory capacity and produce cognitive problems in the short, medium, and long term [23]. Our findings confirm this pattern. Specifically, the students who consumed tobacco, alcohol and other substances, namely cocaine, hashish, LSD, and glue, obtained worse academic results than those who did not consume them. In addition, abstinence from using tobacco, alcohol, and other substances was linked to better academic performance in general, and early exposure to these substances was linked to worse academic results. Similar observations were made by other authors [20,21], who confirmed that academic performance was negatively affected by use of substances. However, other studies [22] did not establish a clear association between tobacco and alcohol consumption and academic performance.

## 5. Limitations

We wish to highlight that this is a cross-sectional study and that, therefore, a clear causal relationship cannot be established between the study variables. Although the response rate was low (34%), the high volume of the sample would allow the results to be externalized, although perhaps by selecting only 43 centers the sample was not completely homogeneous.

## 6. Conclusions

Based on our findings, we argue that there is an association between lifestyle habits and academic performance in adolescents. We observed that those students who maintained a healthy lifestyle obtained better academic results, and those who did not performed worse academically. For this reason, we suggest that health education and promotion programs are implemented in schools to not only promote healthy lifestyles and improve health outcomes among the student population, but also to improve their academic performance. Further, health education and promotion of healthy lifestyles should be integrated into the educational strategies and policies of all educational centers. HPS are an example of a multifactorial intervention that actively works to promote health and healthy lifestyles. There is a solid knowledge-based underpinning the range of interventions and activities implemented by HPS, which should result in improved health outcomes [1,5,42], progressive development of social, emotional, and cognitive abilities and improved academic performance in this population [43]. It is necessary to carry out longitudinal studies in adolescents to reinforce these findings and begin to fill the knowledge gaps identified in this research.

## Figures and Tables

**Table 1:** Relationship between dietary habits and academic performance.

Correlation			
	Kendall Rank Correlation Coefficient	N	*p*-Value
Breakfast during the week	0.100	1589	<0.001
Fruits	0.147	1586	<0.001
Chips	−0.116	1587	<0.001
Vegetables	0.115	1587	<0.001
Sweets	−0.087	1592	<0.001
Soft Drinks	−0.205	1584	<0.001
Fish	0.079	1588	<0.001
Milk and Cow’s milk derivatives	0.086	1588	<0.001
**Hypothesis contrast**			
	Interquartile range	N (Average range)	Contrast test: H (Degree of freedom); *p*
Breakfast during the week Yes No	6.80 (1.96) 6.40 (1.94)	1193 (828.34) 396 (694.56)	196, 441.500 *; <0.001
Fruit Up to once/week 2–6 times/week At least once a day	6.20 (1.75) 6.65 (1.90) 7 (2.02)	251(656.71) 735 (769.19) 600 (880.51)	46.12 (2); <0.001
Chips Up to once/week 2–6 times/week At least once a day	6.95 (2.0) 6.55 (1.84) 6.28 (2.04)	752 (854.40) 705 (758.12) 129 (361.86)	33.54 (2); <0.001
Vegetables Up to once/week 2–6 times/week At least once a day	6.22 (1.70) 6.75 (1.95) 7.05 (2.19)	241 (630.55) 1023 (801.93) 323 (890.84)	45.39 (2); <0.001
Sweet Up to once/week 2–6 times/week At least once a day	6.58 (2) 6.55 (1.87) 6.4 (2.01)	771 (840.09) 623 (773.28) 198 (699.82)	17.28 (2); <0.001
Soft drinks Up to once/week 2–6 times/week At least once a day	7.10 (2.05) 6.3 (1.95) 6 (1.21)	920 (896.58) 532 (665.65) 132 (578.34)	117.50 (2); <0.001
Fish Up to once/week 2–6 times/week At least once a day	6.44 (1.85) 6.9 (1.97) 6.63 (2.39)	633 (723.86) 837 (850.86) 118 (773.65)	27.9 (2); <0.001
Milk and dairy products Up to once/week 2–6 times/week At least once a day	6.45 (2.01) 6.55 (1.83) 6.8 (2.05)	182 (720.17) 516 (742.77) 890 (839.99)	19.99 (2); <0.001

* U Mann–Whitney test.

**Table 2:** Relationship between sleep patterns and academic performance.

Correlation			
	Kendall Rank Correlation Coefficient	N	*p*-Value
Sleep during the week	0.073	1602	<0.001
Hypothesis contrast			
	Interquartile range	N (Average range)	Contrast test: H (Degree of freedom); *p*
Hours of sleep during the week Up to 5 h 7–9 h >9 h	644 (175) 6.75 (2) 7 (1.98)	351 (723.36) 1099 (818.68) 152 (857.73)	13.77 (2); 0.001

**Table 3:** Relationship between physical activity and academic performance.

Correlation			
	Kendall Rank Correlation Coefficient	N	*p*-Value
Weekly frequency of physical activity	0.057	1580	<0.001
Hours of physical activity per week	0.073	1569	<0.001
Team physical activity	0.024	1524	<0.001
Individual physical activity	0.116	1546	<0.001
**Hypothesis contrast**			
	Interquartile range	N (Average range)	Contrast test: H (Degree of freedom); *p*
Weekly frequency of physical activity Never–once a month 1–3 times per week >4 days per week	6.65 (1.98) 6.8 (1.96) 6.2 (1.68)	667 (770.26) 828 (839.85) 107 (699.48)	13.936 (2); 0.001
Hours of physical activity per week Up to 1 h 2–3 h >4 h	6.55 (2.08) 6.67 (1.95) 6.9 (1.83)	629 (767.14) 541 (800.51) 432 (852.77)	8.778 (2); 0.012
Individual physical activity Never–3 times a month 1–3 times a week	6.45 (1.94) 6.8 (2)	704 (741.65) 898 (848.42)	273,958.5 *; <0.001

* U Mann–Whitney test.

**Table 4:** Relationship between physical activity and academic performance.

Correlation				
	Kendall Rank Correlation Coefficient	N	*p*-Value	
Playing videogames Weekdays Weekend	−0.060 −0.126	1582 1580	0.001 <0.001	
Watching TV, videos Weekdays Weekend	−0.108 −0.102	1587 1582	<0.001 <0.001	
Homework and social media Weekdays Weekend	−0.063 −0.090	1582 1579	0.001 <0.001	
**Hypothesis contrast**				
	Interquartile range	N (Average range)		Contrast test: H (Degree of freedom); *p*
Videogames (weekdays) <2 h 2 h >2 h	6.85 (2.15) 6.76 (1.90) 6.5 (1.78)	617 (821.61) 530 (811.64) 435 (724.26)		13.14 (2); 0.001
Videogames (weekend) <2 h 2 h >2 h	6.9 (2.40) 7.05 (2.05) 6.55 (1.82)	224 (829.81) 416 (874.79) 940 (743.83)		25.7 (2); 0.001
Watching TV, videos (weekdays) <2 h 2 h >2 h	7 (2.20) 6.66 (1.80) 6.35 (1.90)	568 (868.31) 672 (783.81) 347 (692.09)		32.42 (2); <0.001
Watching TV, videos (weekend) <2 h 2 h >2 h	6.94 (2.28) 6.95 (2.05) 6.55 (1.80)	202 (844.43) 546 (850.29) 834 (740.19)		22.28 (2); <0.001
Homework; social media (weekdays) <2 h 2 h >2 h	6.85 (2.05) 6.58 (1.95) 6.4 (1.86)	746 (834.38) 514 (783.47) 322 (704.97)		18.28 (2); <0.001
Homework; social media (weekend) <2 h 2 h >2 h	6.85 (2.10) 6.95 (1.95) 6.45 (1.85)	482 (836.59) 463 (839.77) 634 (718.23)		26.26 (2); <0.001

**Table 5:** Relationship between substance use and academic performance.

Correlation			
	Kendall Rank Correlation Coefficient	N	*p*-Value
Tobacco	−0.108	1588	<0.001
Alcohol Wine Mixed drinks Liquor shot Other	−0.088 −0.131 −0.131 −0.108	1518 1521 1515 1519	<0.001 <0.001 <0.001 <0.001
Drugs Hashish	−0.075	1596	<0.001
Age of onset Alcohol Binge-drinking Tobacco Cannabis	−0.182 −0.110 −0.171 −0.086	1594 1595 1592 1591	<0.001 <0.001 <0.001 <0.001
**Hypothesis contrast**			
	**Interquartile range**	**N** **(Average range)**	**Contrast test:** **H (Degree of freedom); *p***
Tobacco No Yes	6.75 (1.95) 5.85 (1.65)	676 (357.91) 28 (221.79)	5804 *; <0.001
Wine Never Rarely Daily-monthly	6.75 (1.94) 6.45 (2.07) 5.04 (2.76)	789 (435.16) 66 (325.20) 12 (217.67)	20.945 (2); <0.001
Mixed drinks Never Rarely Daily-monthly	6.5 (1.76) 6.18 (1.80) 5.39 (1.72)	571 (347.39) 77 (277.60) 21 (208.55)	18.129 (2); <0.001
Liquor shot Never Rarely Daily-monthly	6.76 (1.95) 5.95 (1.81) 5.72 (1.77)	606 (343.54) 50 (260.93) 13 (221.81)	12.987 (2); 0.002
Other alcoholic beverages Never Rarely Daily-monthly	6.75 (1.95) 6.33 (1.84) 5.76 (1.75)	585 (345.03) 65 (270.30) 19 (247.47)	12.759 (2); 0.002
Cocaine Never At least once	6.7 (1.95) 5.98 (1.31)	1590 (803.98) 12 (472.50)	5592 *; 0.013
Hashish Never At least once	6.72 (1.95) 5.83 (1.09)	1572 (807.53) 30 (485.58)	14,102.5 *; <0.001
LSD Never At least once	6.7 (1.95) 6.13 (2.05)	1593 (802.41) 9 (640.44)	5719 *; 0.029
Glue Never At least once	6.7 (1.95) 6 (1.88)	1581 (803.58) 21 (645.05)	13,315 *; 0.019
Age at tobacco use onset Never Less than 11 to more than 14 Age at alcohol use onset Never Before 13 years After 13 years Age at first binge-drinking episode Never Before 13 After 13 years Age at marijuana or hashish use onset Never 11–14	6.8 (1.92) 5.44 (1.55) 6.9 (1.95) 6.44 (1.90) 5.9 (1.65) 6.75 (1.95) 6 (1.88) 5.9 (1.55) 6.75 (1.95) 5.55 (1.85)	1478 (779.07) 53 (401.56) 1190 (861.98) 225 (680.35) 187 (562.41) 1461 (821.65) 47 (621.80) 94 (578.23) 1561 (787.40) 9 (456)	19,851.5; <0.001 * 85.731 (2); <0.001 31.762 (2); <0.001 4059 (0.029) *

* U Mann–Whitney test.

## Data Availability

Data are available contacting with corresponding author.
